# Supplementary material for: National quantifications of methane emissions from fuel exploitation using high resolution inversions of satellite observations
Source: Nat Commun. 2023 Aug 16;14:4948. doi: 10.1038/s41467-023-40671-6 (PMC10432515; doi:10.1038/s41467-023-40671-6)
Supplement: Supplementary file 1 — Supplementary Information [file 41467_2023_40671_MOESM1_ESM.pdf]

Supplementary Information for

Worldwide inference of national methane emissions from fossil fuel exploitation using high-resolution inversions of satellite data

Lu Shen, Daniel J. Jacob, Ritesh Gautam, Mark Omara, Tia R. Scarpelli, Alba Lorente, Daniel Zavala-Araiza, Xiao Lu, Zichong Chen, Jintai Lin

This document contains additional information used to inform our analyses. The following notes, figures, and tables are included:

- **Supplementary Notes 1-2**
- **Supplementary Figures 1-19**
- **Supplementary Tables 1-8**

Supplementary Note 1. Partitioning sectoral emissions

Because CH_4 is emitted by a variety of sources that are collocated, top-down CH_4 measurements estimate the combined methane flux of a source region. Here we partition the fossil fuel emissions through the procedure described by Shen et al. [1], which is written as:

$$f_i = \frac{\eta \alpha_i \sigma_{i,nation}^2 (f_0 - 1)}{\sigma_0^2} + 1 \qquad (1 \leq \mathrm{i} \leq \mathrm{M}) \qquad (S1)$$

$$\eta = \frac{\sigma_0^2}{\sum_{i=1}^{M} \alpha_i^2 \sigma_{i,nation}^2} \qquad (S2)$$

Where α_i is the fraction of emissions of each sector taken from the prior and f_i is the posterior correction factor for i^{th} sector in this gridcell, f_0 is the posterior scaling factors, σ_0 is the prior error standard deviation, M is the number of source sectors. The $\sigma_{i,nation}$ refers to one error standard deviations on the national totals, which are 20% for fossil fuel emissions, 10-30% for other anthropogenic sources and 70% for wetlands [2,3]. The logic here is to make more adjustments for a specific sector if this sector has a higher percentage in prior emissions and higher prior uncertainty.

Supplementary Note 2. Seasonal posterior correction factors and effects of data density at high latitudes

We also calculated posterior emissions from fossil fuel exploitation using TROPOMI observation in different seasons at northern high-latitudes, where the observation density varies considerably across one year (Supplementary Fig. 8-9). In these high-latitude countries, posterior corrections are nearly zero in winter because of scarce observations and low averaging-kernel sensitivities (Supplementary Fig. 16). This result also demonstrates that seasonal corrections are partly influenced by satellite data availability, which does not necessarily reflect the temporal variability in emissions in regions with uneven observation density. In this study, we do not try to optimize for higher temporal variability of emissions because uneven and inadequate seasonal sampling frequencies are present in most regions of the world (Supplementary Fig. 8-9).

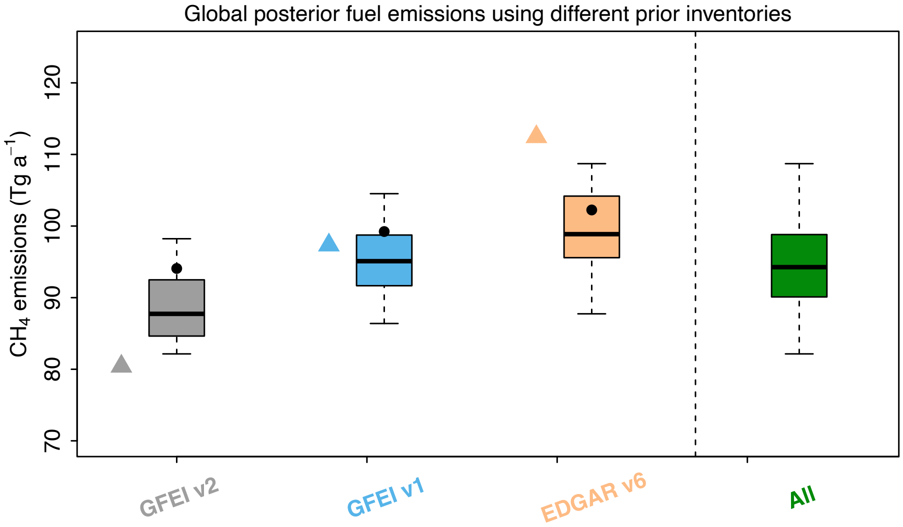

Supplementary Fig. 1. **Global prior vs. posterior methane emissions of fossil fuel exploitation using different prior inventories**. Different colors represent the results using different bottom-up priors and assumptions of error statistics. Triangles denote the magnitude of the priors, points refer to estimates assuming lognormal errors, and boxplots denote the distribution of the posteriors (see Methods for more details).

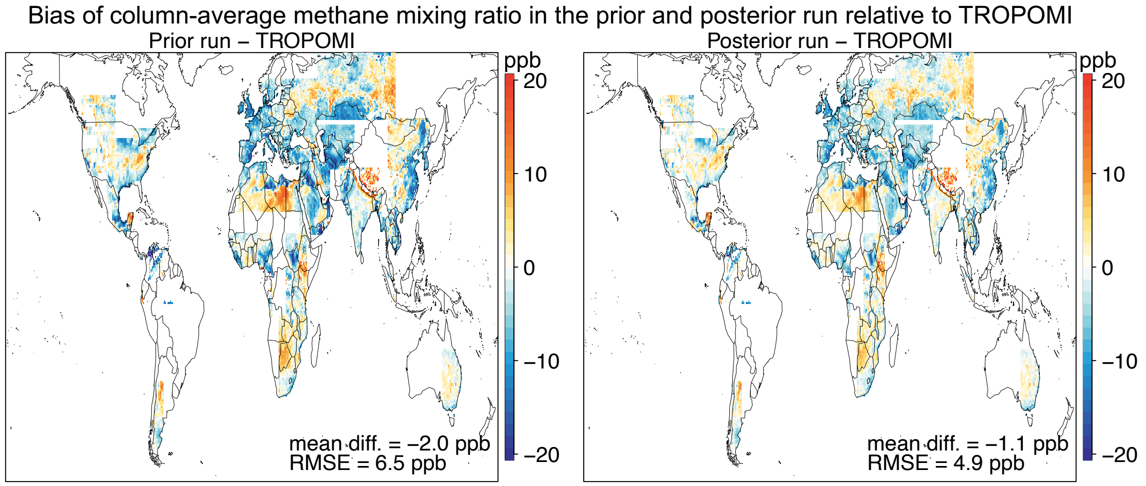

Supplementary Fig. 2. **Bias of column-averaged methane mixing ratio in the prior and posterior GEOS-Chem simulations relative to TROPOMI from May 2018 to February 2022**. To be consistent with our inversion setups, we run the GEOS-Chem model in 15 inversion domains using prior and posterior inventories at the 0.5°×0.625° resolution, and then aggregate the simulation results. The mean bias and root mean square errors (RMSE) are shown inset. The basemap is from the mapdata package (version 2.3.1) in R (https://cran.r-project.org/web/packages/mapdata/index.html).

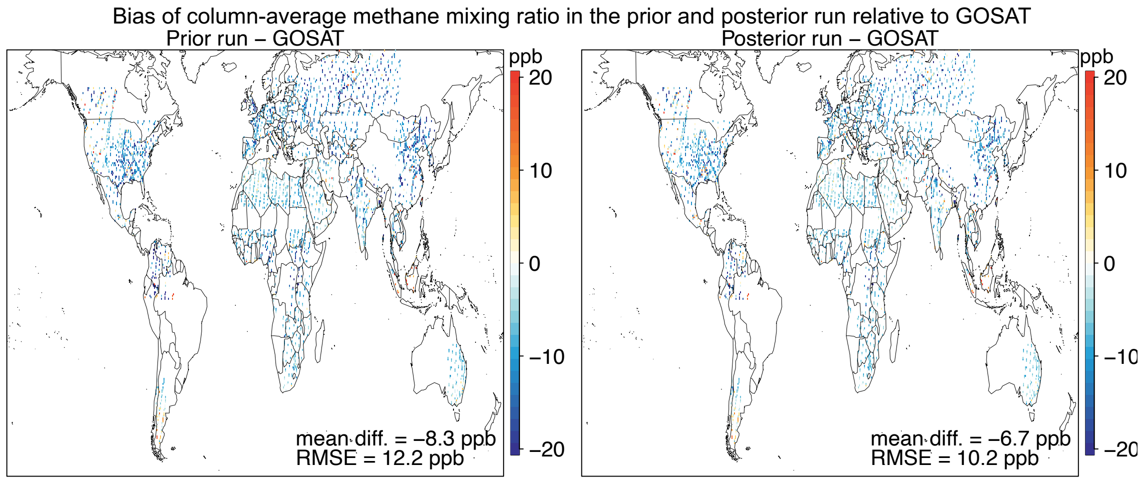

Supplementary Fig. 3. **Bias of column-averaged methane mixing ratio in the prior and posterior GEOS-Chem simulations relative to GOSAT from May 2018 to February 2022**. To be consistent with our inversion setups, we run the GEOS-Chem model in 15 inversion domains using prior and posterior inventories at the 0.5°×0.625° resolution, and then aggregate the simulation results. The mean bias and root mean square errors (RMSE) are shown inset. The basemap is from the mapdata package (version 2.3.1) in R (https://cran.r-project.org/web/packages/mapdata/index.html).

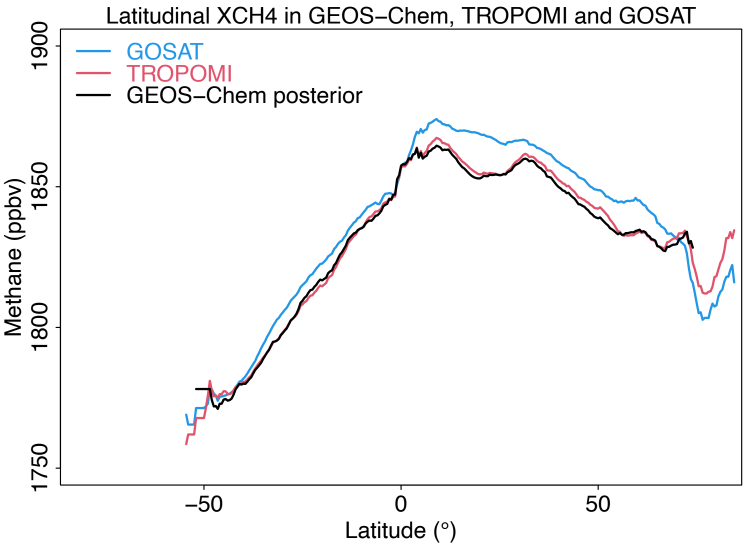

Supplementary Fig. 4. **Latitudinal column-averaged methane mixing ratio between GOSAT, TROPOI, GEOS-Chem posterior runs**. GEOS-Chem simulations are performed in 15 inversion domains with boundary conditions calibrated to match TROPOMI (same as our inversion setups). Thus, GEOS-Chem's latitudinal variability closely resembles TROPOMI's.

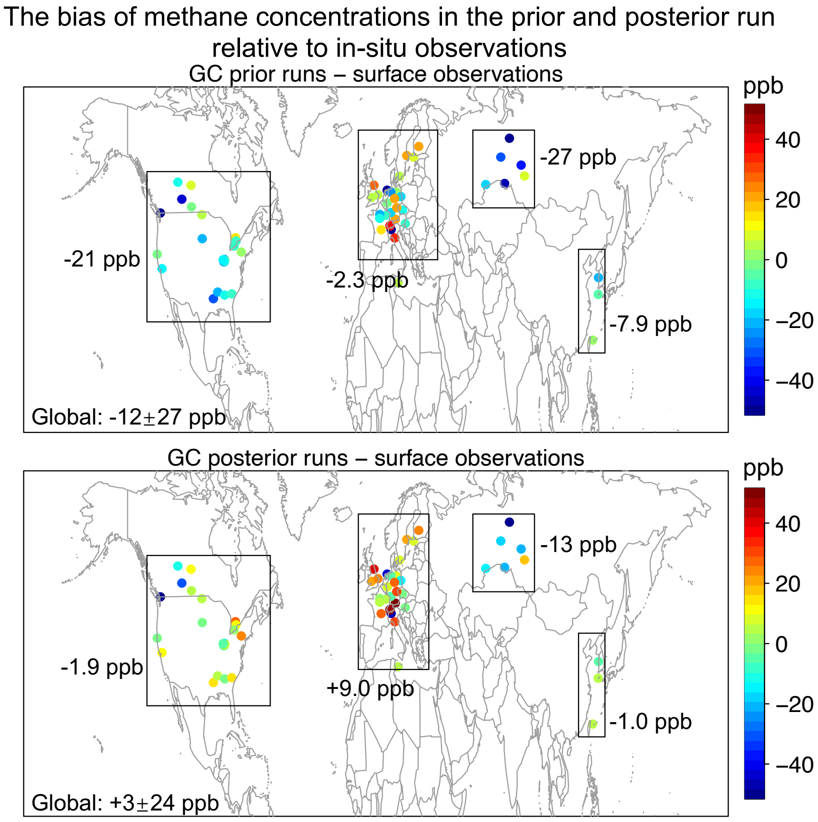

Supplementary Fig. 5. **Bias of surface methane concentrations in the prior and posterior GEOS-Chem simulations** relative to NOAA in-situ observations inside our 15 inversion domains from May 2018 to February 2022 (ObsPack, Schuldt et al.[4]). The mean bias and root mean square errors (RMSE) are shown inset. The basemap is from the mapdata package (version 2.3.1) in R (https://cran.r-project.org/web/packages/mapdata/index.html).

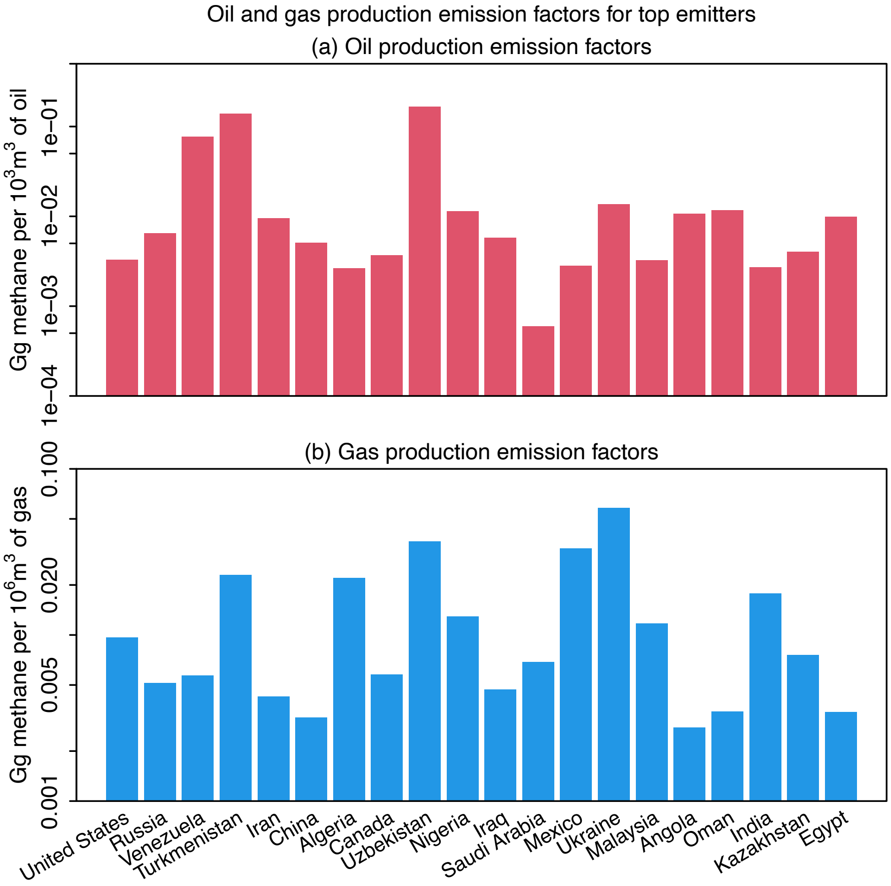

Supplementary Fig. 6. **Methane emission factors for oil and gas production activities in 2019**. The emission factors are calculated as the national methane emissions from the oil and gas sector divided by corresponding production statistics from the EIA (https://www.eia.gov/international/data/world).

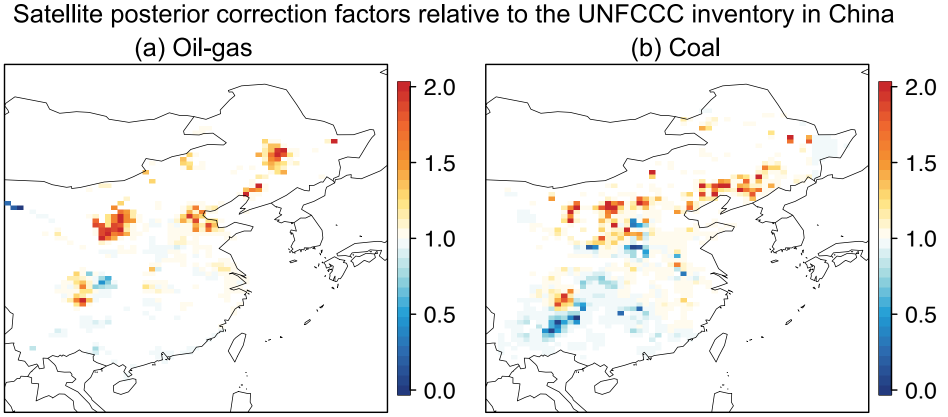

Supplementary Fig. 7. (a) Posterior correction factor from the oil-gas sector relative to the UNFCCC inventory. (b) Same as (a) but for the coal sector. The basemap is from the mapdata package (version 2.3.1) in R (https://cran.r-project.org/web/packages/mapdata/index.html).

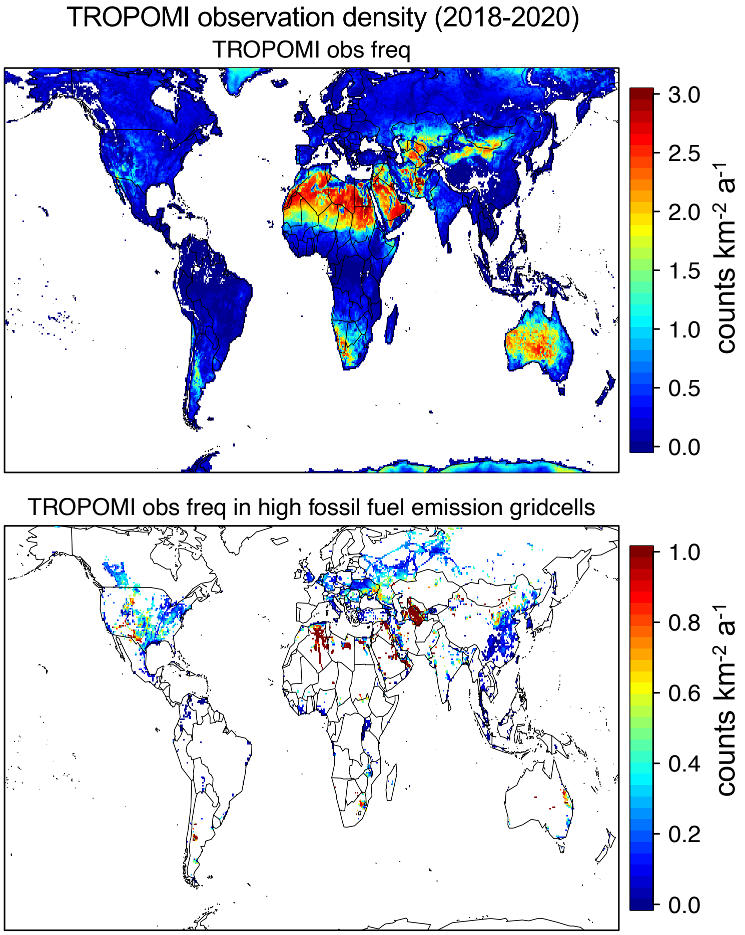

Supplementary Fig. 8. (a) TROPOMI data density (counts km^{-2} a^{-1}) from May 2018 to February 2020, mapped to 0. 5°×0.625° horizontal resolution. (b) Same as (a) but only for gridcells with fossil fuel methane emissions greater than 1 Gg a^{-1}. The basemap is from the mapdata package (version 2.3.1) in R (https://cran.r-project.org/web/packages/mapdata/index.html).

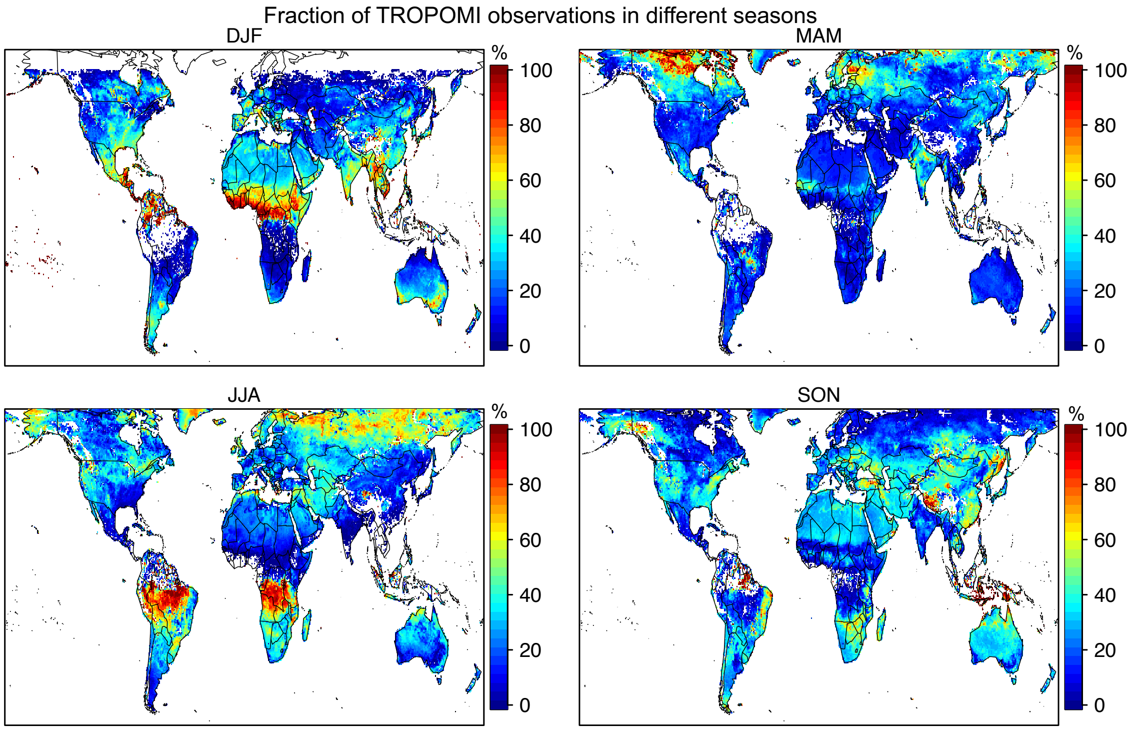

Supplementary Fig. 9. Fraction of TROPOMI observation in different seasons (DJF, MAM, JJA and SON), mapped to 0. 5°×0.625° horizontal resolution. Gridcells with zero observation are shown as white. The basemap is from the mapdata package (version 2.3.1) in R (https://cran.r-project.org/web/packages/mapdata/index.html).

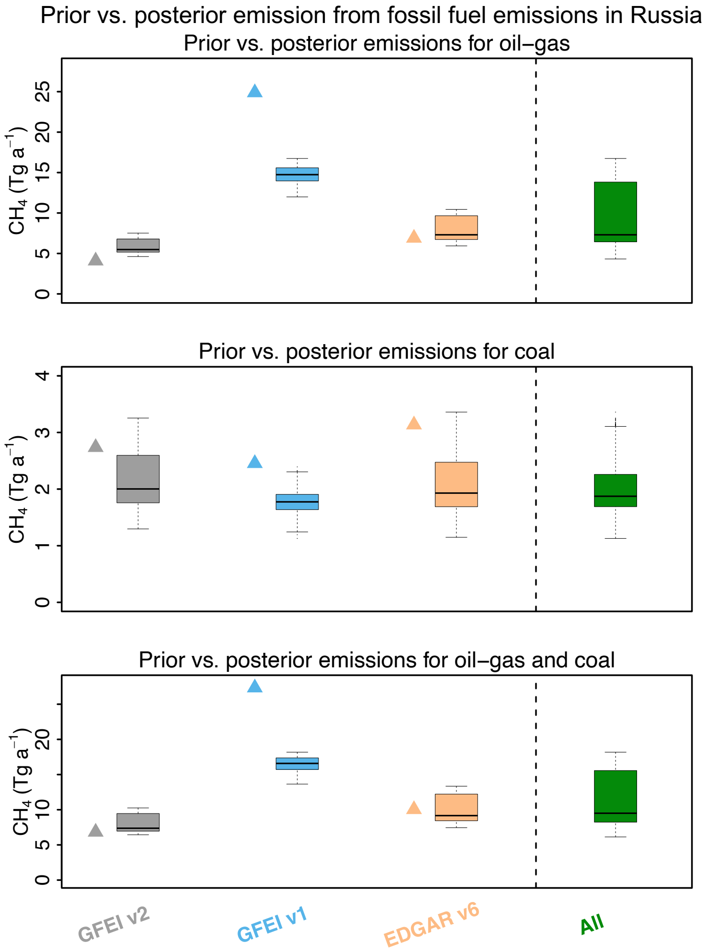

Supplementary Fig. 10. **Prior vs. posterior emissions from the (a) oil-gas, (b) coal, and (c) oil-gas-coal sector in Russia**. Different colors represent the results using different bottom-up priors and assumptions of errors. Triangles denote the magnitude of the priors, and the boxplots denote the distribution of the posteriors.

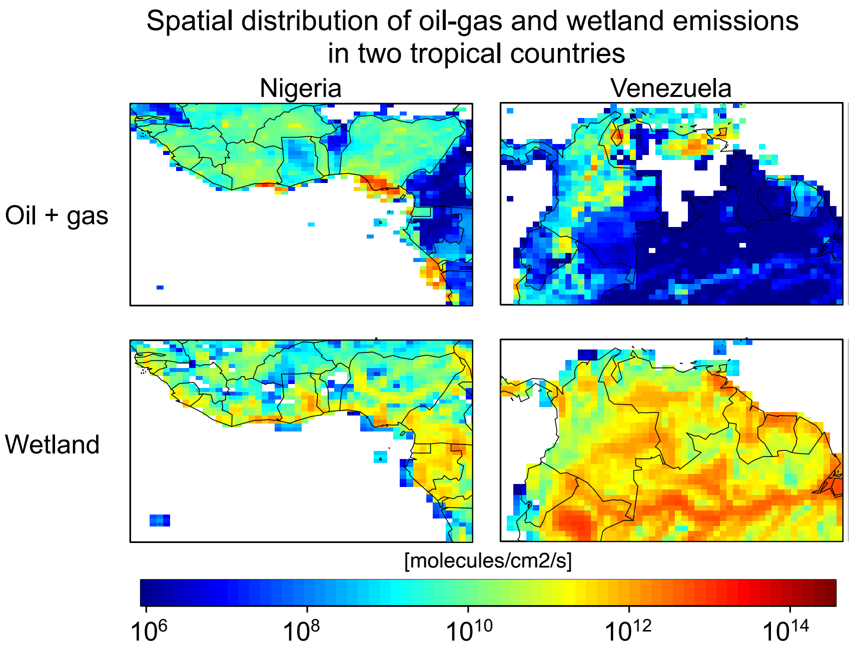

Supplementary Fig. 11. Spatial distribution of oil-gas and wetland emissions in Nigeria and Venezuela. The oil-gas emissions are from GFEI v2, and the wetland emissions are from WetCHARTs v1.3.1[3,5]. The basemap is from the mapdata package (version 2.3.1) in R (https://cran.r-project.org/web/packages/mapdata/index.html).

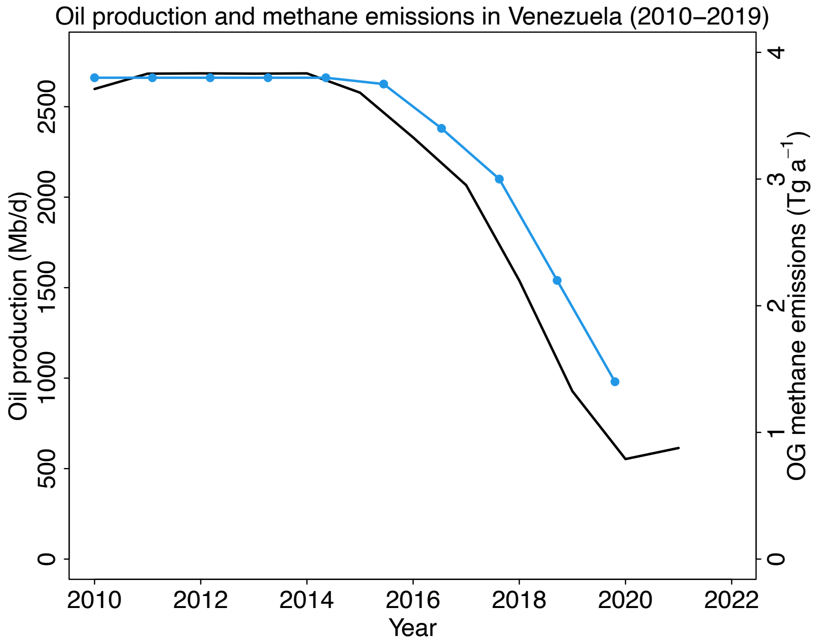

Supplementary Fig. 12. Oil production (https://www.eia.gov/international/data/country/VEN) and fossil fuel (oil + natural gas) methane emissions from GFEIv2 in Venezuela from 2010 to 2019.

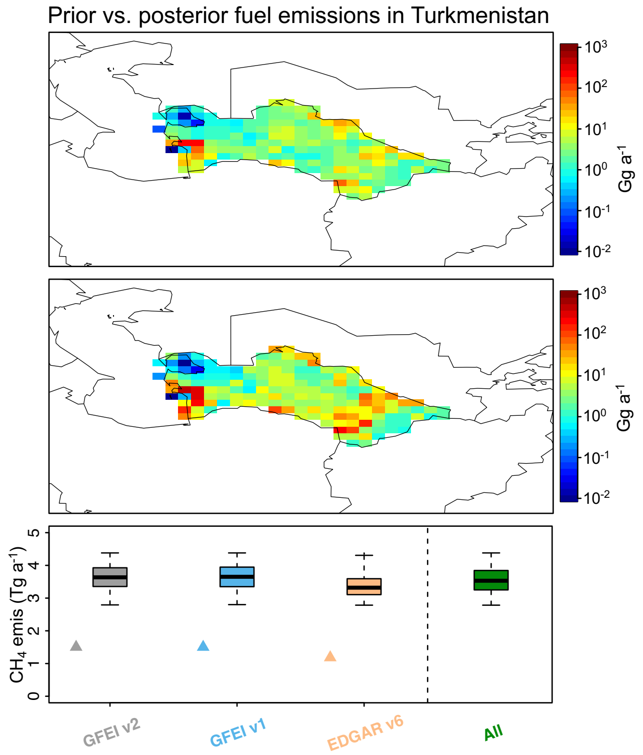

Supplementary Fig. 13. (a) Prior emissions of the fossil fuel industry from GFEI v2 in Turkmenistan. (b) Same as (a) but for the posterior emissions. (c) Prior vs. posterior emissions using different bottom-up inventories and assumptions of errors. Triangles denote the magnitude of the priors, and the boxplots denote the distribution of the posteriors (see Methods for more details). The basemap is from the mapdata package (version 2.3.1) in R (https://cran.r-project.org/web/packages/mapdata/index.html).

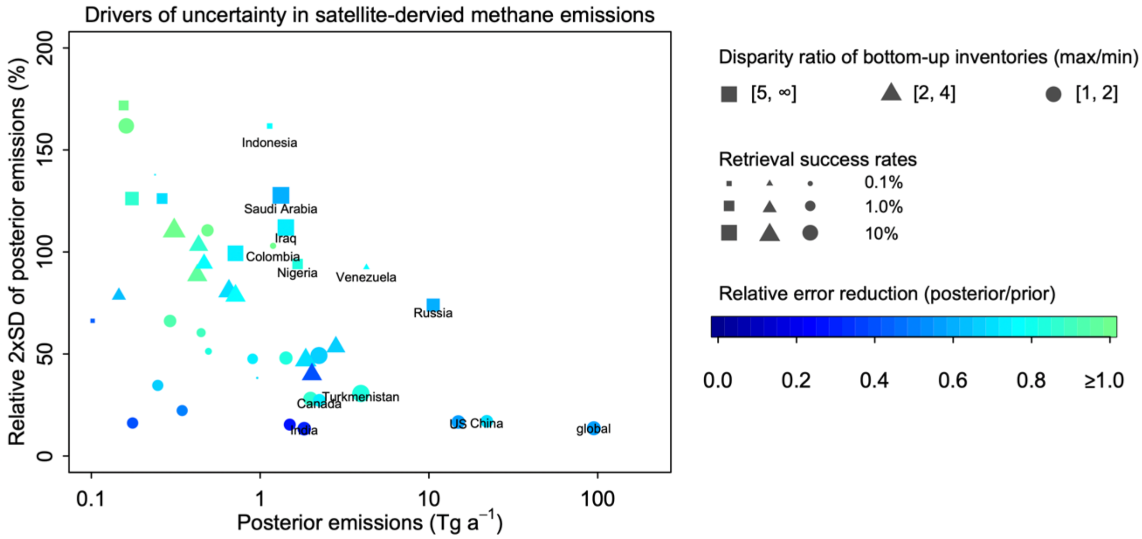

Supplementary Fig. 14. **Drivers of posterior uncertainty of satellite-derived national-scale methane emissions from the fossil fuel industry**. The disparity ratio of bottom-up inventories is defined as the ratio of maximum to minimum emissions from GFEI v2, GFEI v1, and EDGAR v6, and different symbols (squares, triangles, and circles) denote the magnitudes. The symbol size denotes TROPOMI retrieval success rates, calculated as the percentage of high-quality retrievals relative to the total number of satellite pixels in each

0.5°×0.625° gridcell. Colors refer to the ratio of the relative posterior SD to the prior one. Only countries with total fossil fuel emissions > 0.1 Tg a^{-1} are shown here.

Effects of wetland emissions on posterior methane emissions from fossil fuel exploitation

(a) WetCHARTs wetland methane emissions (high performance – all ensembles)

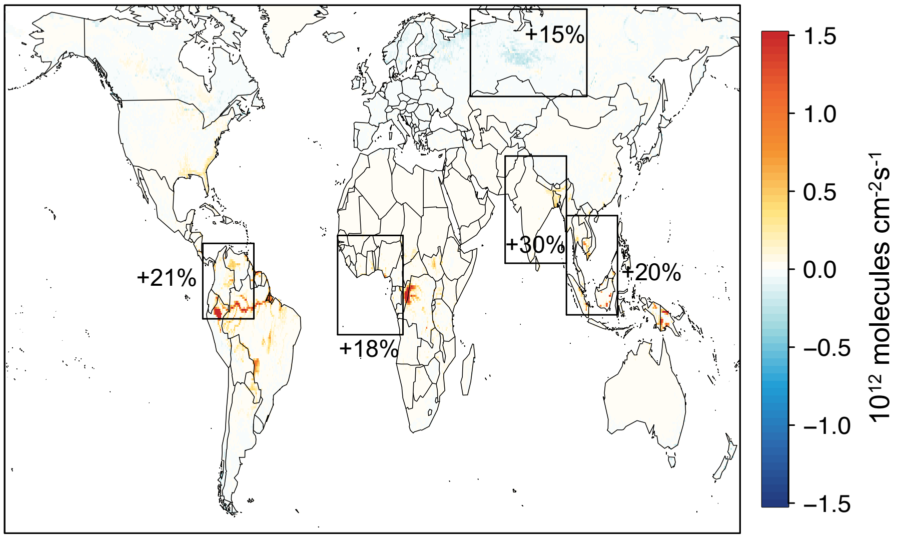

(b) Δmethane emissions from fossil fuel exploitation in the top 5 countries (high performance – all ensembles)

	Venezuela	Russia	India	Nigeria	Cote d'Ivoire
Posterior OG and Coal emissions using high performance WetCHART members (Tg a^{-1})	4.2	11.3	1.9	3.4	0.27
Δemissions if using all WetCHART members (Tg a^{-1})	-0.7	0.3	-0.2	-0.15	-0.15

Supplementary Fig. 15. Effects of wetland inventories on posterior fossil fuel emissions. (a) Difference in wetland methane emissions using the 9 highest-performance members and 18 all members in WetCHARTs v1.3.1[3,5]. The five inversion domains with the highest relative difference in wetland methane emissions are shown as black rectangles. (b) The 5 top countries with the highest absolute changes of posterior methane emissions from fossil fuel exploitation if using all 18 WetCHARTs members. The basemap is from the mapdata package (version 2.3.1) in R (https://cran.r-project.org/web/packages/mapdata/index.html).

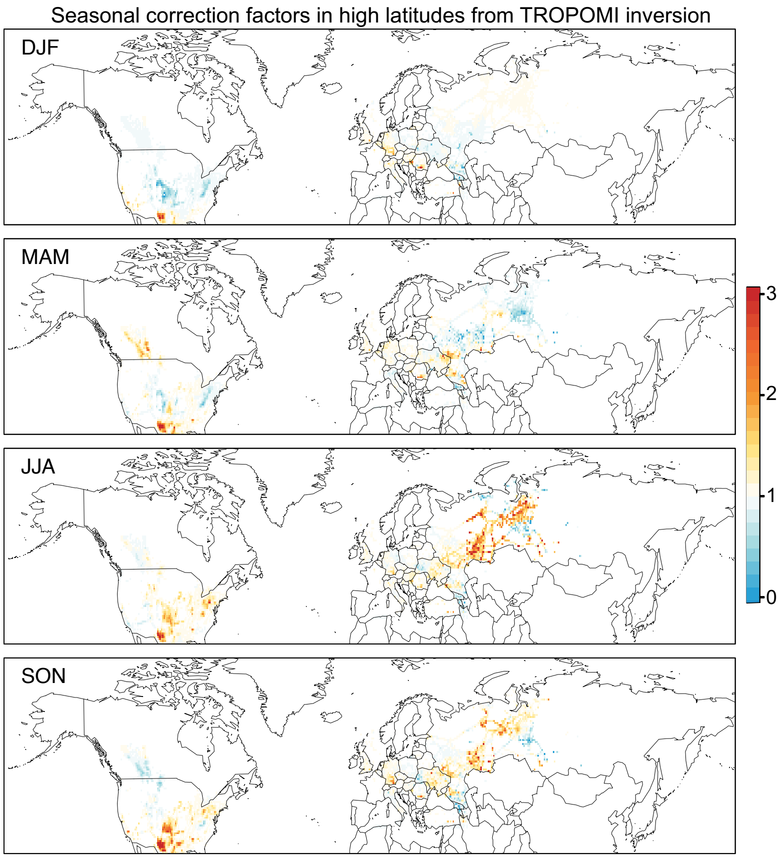

Supplementary Fig. 16. Posterior correction factors in different seasons at high latitudes north of 30°N. This test uses GFEIv2 as the prior inventory. Please note that the seasonal corrections here are largely determined by the sampling frequency in different seasons (Supplementary Fig. 8-9), which do not necessarily reflect the temporal variability in emissions. See Supplementary Note 2 for discussion. The basemap is from the mapdata package (version 2.3.1) in R (https://cran.r-project.org/web/packages/mapdata/index.html).

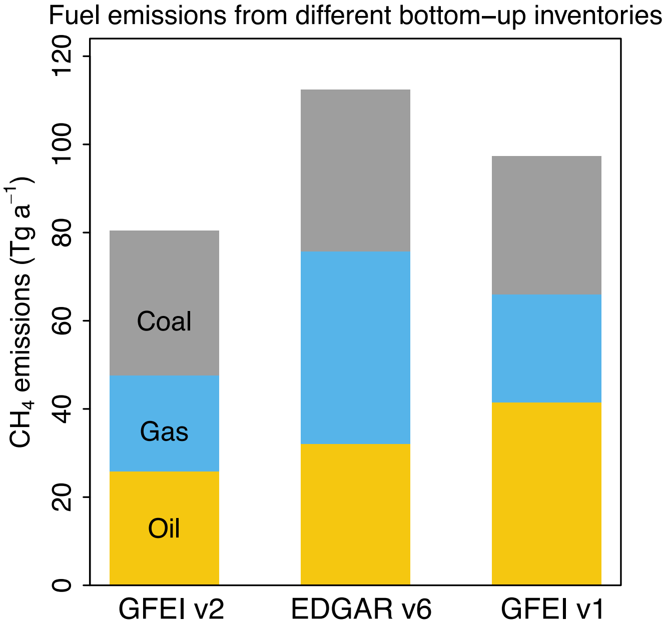

Supplementary Fig. 17. Methane emissions from different bottom-up inventories as well as the composition in 2019.

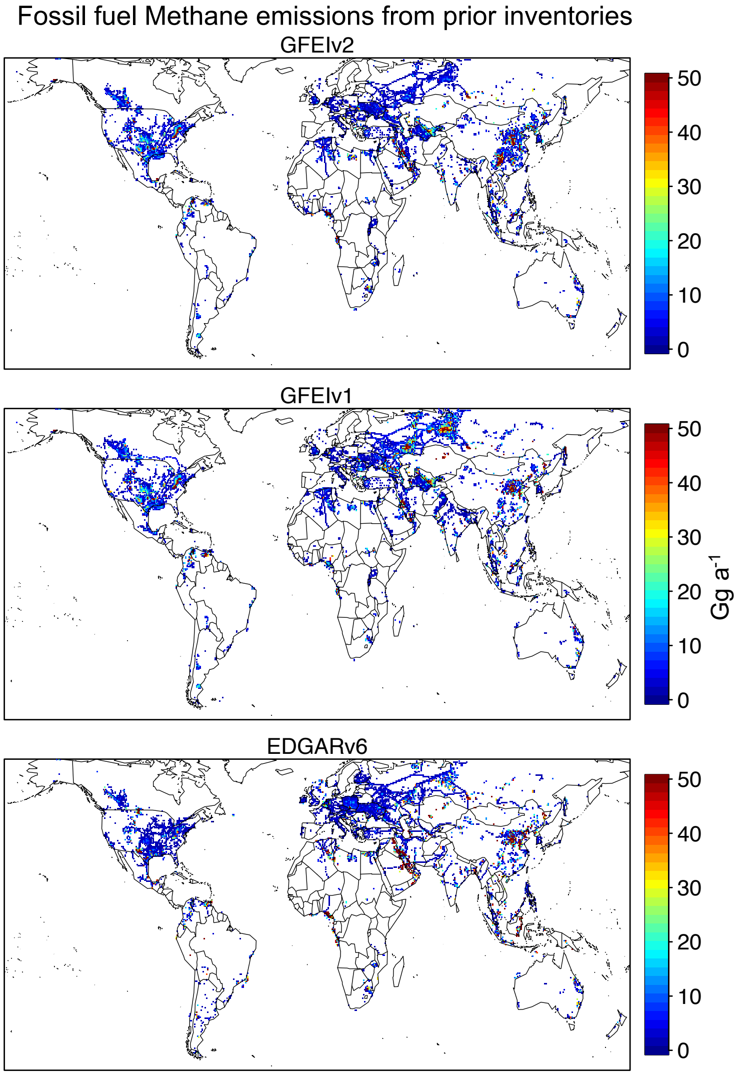

Supplementary Fig. 18. Spatial distribution of fossil fuel methane emissions from GFEIv2, GFEIv1 and EDGARv6. We only show those gridcells with emissions greater than 1 Gg a^{-1}. The basemap is from the mapdata package (version 2.3.1) in R (https://cran.r-project.org/web/packages/mapdata/index.html).

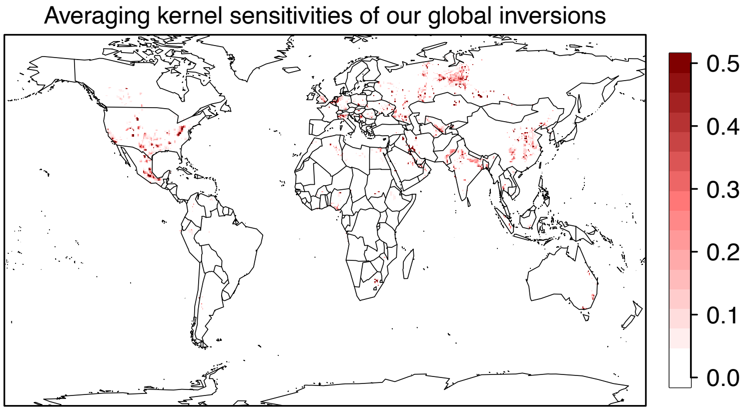

Supplementary Fig. 19. Averaging kernel sensitivities representing the diagonal terms of the averaging kernel matrix. The total degree of freedoms (DOFS) is 568. The basemap is from the mapdata package (version 2.3.1) in R (https://cran.r-project.org/web/packages/mapdata/index.html).

Supplementary Table 1. Details of field-campaigns from recently published data displayed in Figure 2.

Countries	Basin names	Method	location	Year	Reference
**Australia**	Surat	Airborne measurements, mass balance	27.0°S, 150.5°E	2018	[6]
**Germany**	Ibbenburen	Airborne remote sensing instrument, Gaussian plume inversion	52.3°N, 7.7°E	2011	[7]
**Netherland**	Gronigen	Airborne measurements, mass balance	53.1°N, 6.3°E	2016	[8]
**Poland**	Upper Silesian Coal Basin	Aircraft- and ground-based observations, mass-balance	50.1°N, 18.7°E	2018	[9]
**Canada**	Albert west	Ground-based and aircraft measurements, Gaussian dispersion method	52.4°N, 114°W	2016	[10,11]
	Lloydminster	Airborne measurements, mass balance	53.8°N, 110.5°W	2016	[11]
**US**	Bakken2014	Airborne measurements, mass balance	47.5°N, 102.5°W	2014	[12]
	Bakken2015	Airborne measurements, mass balance	47.8°N, 102.5°W	2015	[13]
	Barnett	Airborne measurements, mass balance	32.9°N, 97.3°W	2013	[14]
	Delaware	Tower and aerial measurements	31.9°N, 103.7°W	2020	[15]
	Denver Basin	Airborne measurements, mass balance	40.3°N, 104.7°W	2015	[13]
	Denver-Julesburg	Airborne measurements, mass balance	39.9°N, 104.5°W	2012	[16]
	Eagle Ford East	Airborne measurements, mass balance	28.9°N, 98°W	2015	[13]
	Eagle Ford West	Airborne measurements, mass balance	28°N, 99.5°W	2015	[13]
	Fayetteville	Airborne measurements, mass balance	35.3°N, 92.2°W	2015	[17]
	Haynesville-Bossier	Airborne measurements, mass balance	32.2°N, 94.0°W	2013	[18]
	Haynesville	Airborne measurements, mass balance	32.2°N, 94.2°W	2015	[13]
	Marcellus	Airborne measurements, mass balance	41.6°N, 76.8°W	2013	[13]
	NE PA	Airborne measurements, mass balance	41.8°N, 76.5°W	2015	[19]
	Permian	Ground measurements	32°N, 103°W	2018	[20]
	San Juan	Airborne measurements, mass balance	36.6°N, 107.7°W	2015	[21]
	SW PA	Airborne measurements, mass balance	39.8°N, 80.2°W	2015	[22]
	Uinta	Ground measurements, basin-constrained emission estimate	40.1°N, 109.8°W	2015-2016	[23]
	Western Arkoma	Airborne measurements, mass balance	35.4°N, 93.7°W	2013	[24]

Supplementary Table 2. Estimates from field campaigns are compared to results from our TROPOMI inversions. We adjust the TROPOMI-based emission estimates# at the basin scale to field campaign years using the 2010-2019 relative linear trends from Fig. 2b in [25]. The emission trends in Lu et al. are obtained using high-resolution inversion of surface and GOSAT observations in North America.

		Field campaign		TROPOMI (Gg a^{-1})		Description
**Countries**	**Basin names**	**Emissions** (Gg a^{-1})	**Data sources** (see Supplementary Table 1 for references)	**Inversion**	**Scaled to field campaign years#**	
**Australia**	Surat	63	Fig. 1 (domain), Fig. 3 (emissions, top-down and UNSW decomposition)	28		
**Germany**	Ibbenburen	50	Fig. 1 (domain), Abstract (emissions)	77		We only use the inversion results using EDGARv6 as the prior because GFEIv1 and GFEIv2 have zero coal emissions in this campaign location.
**Netherland**	Gronigen	14	Fig. 1 (domain), Abstract (emissions, 20% from OG)	4		
**Poland**	Upper Silesian Coal Basin	396	Fig. 2 (domain), Section 4 (emissions, 87% from coal from CAMS inventory)	264		
**Canada**	Albert west	35	Fig. 1 (domain), Page 8 (emissions)	80	114	
	Lloydminster	210	Table 1 (domain), Fig. 5 (emissions)	88	133	
**US**	Bakken2014	240	Fig. 1 (domain), Table 2 (emissions)	155	190	
	Bakken2015	245	Fig. S1 (domain), Table 1 (emissions)	155	183	
	Barnett	530	Fig. 1 (domain), Abstract (emissions)	705	788	
	Delaware	1440	Fig. 1 (domain), Table 1(emissions, using pre-crash, July and August values, and 98% of emissions from OG)	850	850	This inversion uses the GEPA inventory as the prior, in which Permian's emission is 620 Gg a^{-1} in 2019[26].
		1440	Same as above	970	970	Permian's prior emission is scalled to 2500 Gg a^{-1} to match EDF's inventory[20].
	Denver Basin	158	Fig. S3 (domain), Table 1 (emissions)	31	69	
	Denver-Julesburg	169	Fig. 1 (domain), Abstract (emissions)	51	222	
	Eagle Ford East	370	Fig. 3 (domain), Table 1 (emissions)	396	333	
	Eagle Ford West	359	Fig. 2 (domain), Table 1 (emissions)	203	171	
	Fayetteville	238	Fig. 1 (domain), Table 1 (emissions)	36	47	
	Haynesville-Bossier	426	Fig. 5 (domain), Abstract (emissions),	688	550	

			67% is from OG based on our prior inventories			
	Haynesville	368	Fig. S4 (domain), Table 1 (emissions)	643	545	
	Marcellus	114	Fig. 9 (domain), Table 2 (emissions)	58	57	
	NE PA	131	Fig. 3 for domain and Alvariez for emissions	84	83	
	Permian	2660	Methods in Zhang et al. [27] (2.3 Tg for OG production, 0.22 Tg from compressor plants, and 0.14 Tg from processing plants)	2900	2900	This inversion uses the GEPA inventory as the prior, in which Permian's emission is 620 Gg a^{-1} in 2019[26].
		2660	Same as above	3700	3700	Permian's prior emission is scalled to 2500 Gg a^{-1} to match EDF's inventory[20].
	San Juan	440	Fig. 1 (domain), Section 4.3.4 (emissions)	284	710	
	SW PA	190	Fig. 2 (domain), Abstract (emissions)	38	38	
	Uinta	270	Fig. 1 (domain), Abstract (emissions, assuming 59% from OG based on our prior inventories)	145	218	
	Western Arkoma	228	Fig. 7 (domain), Table 2 (emissions)	62	93	

Supplementary Table 3. Methane emissions from the oil-gas and coal exploitation in different top-down studies. The uncertainty in Saunois et al. [28,29#] refers to the min-max range of reported studies. Some studies[*] didn't report the uncertainty specifically for the fossil fuel sector; thus, we use the uncertainty of global anthropogenic emissions here. The uncertainties in these studies[*] are extremely low because they assume the grid-scale posterior estimates are independent with each other; thus, the uncertainty of global emissions inversely scales with the length of state vectors.

	Oil and Gas (Tg a^{-1})		Coal (Tg a^{-1})		Fossil fuel (Tg a^{-1})		Years of emissions
Top-down studies	Mean	Uncertainty	Mean	Uncertainty	Mean	Uncertainty	
Saunois et al.[28]					101	77-126[#]	2000-2009
					105	77-133[#]	2003-2012
					112	90-137[#]	2012
Saunois et al.[29]					101	71-151[#]	2000-2009
					111	81-131[#]	2008-2017
					108	91-121[#]	2017
Turner et al.[30]	67	NA	30	NA	97	NA	2009-2011
Zhang et al.[31]	59.4	2%[*]	21	2%[*]	80.4	2%[*]	2010-2018
Lu et al.[32]	70	NA	23	NA	93	NA	2010-2017
Maasakkers et al.[33]	67.5	<2%[*]	27.6	<2%[*]	95	<2%[*]	2010-2015
Qu et al.[34]	54	5%[*]	26	NA	80	5%[*]	2019
Fraser et a.[35]	NA		NA		77.8	71.8-83.8	2009
**This work**	62.6	50.9-73.2	32.6	29.3-37.8	95.4	81.5-108.3	2018-2020

Supplementary Table 4. National methane emissions from the oil-gas and coal sectors. Latest UNFCCC reports as of September 2021 (2019 emissions for Annex I countries) and posterior estimates for May 2018 – February 2020 from inversion of TROPOMI data. Numbers in parentheses are 95% confidence limits on the TROPOMI estimates. Diagonal terms of the averaging kernel matrix for the TROPOMI inversion after summation over the country, representing the number of independent pieces of information on emissions within the country quantified by the inversion.

	Oil gas emissions (Gg a^{-1})		Coal emissions (Gg a^{-1})		Averaging kernel sensitivities
	UNFCCC report	TROPOMI (95% CI)	UNFCCC report	TROPOMI (95% CI)	
Afghanistan	7	5 (3-8)	4	32 (4-95)	0.1
Algeria	1100	2200 (1400-3300)	<1	<1 (0-0)	4.9
Angola	980	910 (550-1400)	<1	<1 (0-0)	1.1
Argentina	360	410 (230-860)	1	<1 (0-1)	1.1
Australia	330	340 (240-440)	900	1600 (1100-2500)	8.4
Belarus	35	27 (15-35)	<1	<1 (0-0)	1.6
Bolivia	49	<1 (0-0)	<1	<1 (0-0)	<0.1
Botswana	9	2 (0-7)	23	<1 (0-13)	0.3
Brazil	180	150 (45-500)	44	44 (44-44)	1.1
Burkina Faso	37	22 (0-41)	<1	<1 (0-0)	1
Burma	11	81 (8-280)	4	3 (1-4)	0.5
Cameroon	14	350 (4-1100)	<1	1 (0-2)	0.9
Canada	1400	2200 (1600-2800)	55	40 (36-64)	11
Central African Republic	9	<1 (0-0)	<1	<1 (0-0)	0.1
Chad	82	<1 (0-0)	<1	<1 (0-1)	0.2
Chile	43	10 (6-15)	2	2 (2-2)	0.7
China	1200	2700 (1900-4100)	21000	19000 (16000-23000)	57
Colombia	270	500 (250-860)	310	580 (130-2000)	1.6
Congo-Brazzaville	11	12 (6-22)	<1	<1 (0-0)	0.7
Congo-Kinshasa	220	230 (16-600)	<1	<1 (0-0)	0.1
Cote d'Ivoire	820	260 (30-630)	<1	<1 (0-0)	1
Cuba	110	<1 (0-0)	<1	<1 (0-0)	<0.1
Ecuador	38	52 (0-140)	<1	<1 (0-0)	0.6
Egypt	390	690 (360-1200)	<1	<1 (0-0)	3.4
Ethiopia	3	1 (0-1)	<1	<1 (0-0)	3
Finland	1	49 (1-160)	<1	<1 (0-0)	0.4
France	44	100 (45-230)	<1	<1 (0-1)	2.9
French Polynesia	<1	<1 (0-0)	<1	<1 (0-0)	<0.1
Gabon	6	130 (6-440)	<1	<1 (0-0)	<0.1
Germany	190	200 (160-240)	6	110 (6-280)	8.1
Ghana	140	74 (23-150)	<1	<1 (0-0)	0.5
Greece	5	29 (4-80)	28	29 (25-33)	<0.1
Guinea	26	17 (6-27)	<1	<1 (0-0)	0.5
Guyana	1	<1 (0-1)	<1	<1 (0-0)	0.1
India	940	640 (390-870)	880	1200 (990-1500)	37
Indonesia	620	600 (380-930)	200	750 (110-2700)	5.1
Iran	3300	2900 (1800-4300)	22	37 (29-43)	12
Iraq	2900	1300 (150-2400)	<1	<1 (0-0)	6.1
Italy	160	180 (160-200)	1	1 (0-2)	7.4
Japan	10	20 (19-22)	31	27 (19-34)	<0.1
Kazakhstan	200	660 (270-1200)	280	1300 (850-2000)	5.2
Kenya	2	1 (0-2)	<1	<1 (0-0)	0.6
Laos	12	7 (0-12)	4	11 (1-39)	<0.1
Libya	800	310 (0-560)	<1	<1 (0-0)	1.7
Madagascar	57	<1 (0-0)	<1	<1 (0-0)	<0.1
Malaysia	980	990 (680-1400)	2	7 (2-19)	0.1
Mali	38	13 (0-40)	<1	<1 (0-0)	0.6

Mauritania	13	<1 (0-0)	<1	<1 (0-0)	<0.1
Mexico	620	1300 (1100-1500)	230	280 (230-320)	32
Mongolia	13	28 (4-98)	50	43 (0-180)	0.8
Morocco	39	46 (10-67)	<1	<1 (0-0)	1.1
Mozambique	8	40 (5-100)	180	130 (19-350)	1.4
Namibia	6	<1 (0-0)	<1	<1 (0-0)	<0.1
New Zealand	16	7 (7-7)	7	7 (7-7)	<0.1
Niger	8	1 (0-1)	<1	<1 (0-0)	0.7
Nigeria	3300	1500 (470-2900)	<1	140 (0-530)	7.9
Norway	14	160 (9-590)	3	2 (1-3)	0.1
Oman	680	700 (120-1300)	<1	<1 (0-0)	1.7
Pakistan	150	420 (140-800)	35	66 (42-110)	10
Papua New Guinea	30	<1 (0-0)	<1	<1 (0-0)	<0.1
Paraguay	2	<1 (0-0)	<1	<1 (0-0)	<0.1
Peru	90	130 (45-270)	11	11 (11-11)	1.9
Philippines	14	58 (35-120)	41	51 (40-74)	<0.1
Poland	130	250 (75-840)	660	250 (53-600)	4.6
Romania	110	310 (140-640)	220	160 (14-400)	3.3
Russia	4100	9400 (4500-17000)	2700	2000 (1400-2900)	140
Saudi Arabia	550	1500 (620-3000)	<1	<1 (0-0)	6.1
Senegal	2	1 (0-1)	<1	<1 (0-0)	0.2
Solomon Islands	1	<1 (0-0)	<1	<1 (0-0)	<0.1
Somalia	23	<1 (0-0)	<1	<1 (0-0)	<0.1
South Africa	69	56 (4-280)	1200	610 (260-1000)	6.6
South Sudan	22	38 (1-140)	<1	<1 (0-0)	1.5
Spain	7	31 (7-63)	1	2 (0-5)	0.2
Sudan	46	120 (45-480)	<1	<1 (0-0)	2.9
Sweden	1	6 (1-15)	<1	<1 (0-0)	0.4
Tanzania	7	24 (7-53)	9	7 (3-10)	0.5
Thailand	510	410 (120-590)	25	20 (11-29)	3.5
Tunisia	37	97 (30-190)	<1	<1 (0-0)	0.3
Turkey	110	130 (110-170)	270	200 (120-280)	0.6
Turkmenistan	1500	3600 (2700-5500)	<1	<1 (0-0)	3.3
Uganda	94	60 (0-120)	<1	<1 (0-0)	0.7
Ukraine	1300	1100 (660-1700)	480	300 (55-720)	12
United Kingdom	180	240 (180-350)	19	16 (0-31)	4.4
United States	8100	13000 (10000-15000)	2100	2600 (2100-3100)	88
Uruguay	<1	<1 (0-0)	<1	<1 (0-0)	<0.1
Uzbekistan	1900	1800 (1200-2300)	6	3 (2-5)	11
Venezuela	1400	4000 (1300-7400)	38	100 (25-530)	1.3
Vietnam	460	290 (120-560)	110	270 (68-540)	1.1
Western Sahara	2	<1 (0-1)	<1	<1 (0-0)	<0.1
Yemen	25	63 (10-160)	<1	<1 (0-0)	0.1
Zambia	1	1 (0-1)	1	1 (0-1)	0.5
Zimbabwe	1	<1 (0-1)	14	9 (2-22)	0.5
global	48000	63000 (51000-73000)	33000	33000 (29000-38000)	570

Supplementary Table 5. Posterior estimates of oil-gas methane emissions (2018-2020) using different priors (GFEIv2, GFEIv1 and EDGARv6) for top emitting countries. For the US and Canada[#], the prior inventory is based on GFEIv2, which has improved spatial distribution relative to GFEIv1 and EDGARv6. We also scale the prior in the Permian basin to match EDF's inventory[20] (extrapolated from site-scale emission rates) in sensitivity experiments, following the same setups in Shen et al.[36]. For Mexico[†], we use GFEIv2 as the prior and scale Mexico's offshore emissions by a factor of 0.1 to match field campaign results[37], following the same setups in Shen et al.[1] .

Countries	using GFEIv2 (UNFCCC)		using GFEIv1		using EDGARv6		Averaged posterior ($Tg\ a^{-1}$)
	Prior ($Tg\ a^{-1}$)	Posterior ($Tg\ a^{-1}$)	Prior ($Tg\ a^{-1}$)	Posterior ($Tg\ a^{-1}$)	Prior ($Tg\ a^{-1}$)	Posterior ($Tg\ a^{-1}$)	
United States[#]	8.1	12.6	8.3		9.6		12.6
Russia	4.1	5.6	24.9	15.0	6.9	7.7	9.4
Venezuela	1.4	4.1	3.2	6.4	0.9	1.6	4.0
Turkmenistan	1.5	3.9	1.5	3.8	1.3	3.1	3.6
Iran	3.3	2.2	4.1	2.6	7.4	3.9	2.9
China	1.2	2.2	1.1	2.1	3.2	3.8	2.7
Algeria	1.1	2.0	1.2	2.1	2.1	2.5	2.2
Canada[#]	1.4	2.2	1.6		1.6		2.2
Uzbekistan	1.9	2.1	2.6	2.1	1.2	1.1	1.8
Nigeria	3.3	2.1	0.4	0.5	3.7	1.9	1.5
Iraq	2.9	1.7	0.1	0.2	4.6	2.0	1.3
Saudi Arabia	0.6	0.8	0.6	1.1	4.7	2.8	1.5
Mexico[†]	0.6	1.3	0.6		0.9		1.3
Ukraine	1.3	1.3	1.1	0.9	0.9	1.1	1.1
Malaysia	1.0	0.9	0.9	0.9	1.3	1.2	1.0
Angola	1.0	0.8	1.1	1.0	1.2	0.9	0.9
Oman	0.7	0.9	0.1	0.3	1.1	0.9	0.7
India	0.9	0.7	1.0	0.8	0.7	0.5	0.6
Kazakhstan	0.2	0.5	0.3	0.6	0.9	0.9	0.7
Egypt	0.4	0.5	0.4	0.5	1.1	1.1	0.7

Supplementary Table 6. Posterior estimates of coal-based methane emissions (2018-2020) using different priors (GFEIv2, GFEIv1 and EDGARv6) for top emitting countries. For the US and Canada[#], the prior inventory is based on GFEIv2, which has improved spatial distribution relative to GFEIv1 and EDGARv6. We also scale the prior in the Permian basin to match EDF's inventory[20] (extrapolated from site-scale emission rates) in sensitivity experiments, following the same setups in Shen et al.[36]. For Mexico[†], we use GFEIv2 as the prior and scale Mexico's offshore emissions by a factor of 0.1 to match field campaign results[37], following the same setups in Shen et al.[1] .

Countries	using GFEIv2 (UNFCCC)		using GFEIv1		using EDGARv6		Averaged posterior ($Tg\ a^{-1}$)
	Prior ($Tg\ a^{-1}$)	Posterior ($Tg\ a^{-1}$)	Prior ($Tg\ a^{-1}$)	Posterior ($Tg\ a^{-1}$)	Prior ($Tg\ a^{-1}$)	Posterior ($Tg\ a^{-1}$)	
China	21.1	20.2	18.5	18.2	19.9	18.2	18.9
United States[#]	2.1	2.6	2.9		1.6		2.6
Russia	2.7	2.0	2.5	1.8	3.1	2.0	2.0
Australia	0.9	1.6	1.0	1.7	0.9	1.6	1.6
Kazakhstan	0.3	1.2	0.9	1.4	0.7	1.4	1.3
India	0.9	1.1	0.8	1.0	1.2	1.4	1.2
Colombia	0.3	0.7	0.3	0.6	0.2	0.4	0.6
South Africa	1.2	0.7	0.4	0.5	1.2	0.7	0.6
Indonesia	0.20	0.19	0.14	0.13	4.51	1.93	0.75
Ukraine	0.48	0.30	0.65	0.36	0.16	0.24	0.30
Mexico[†]	0.23	0.22	0.22		0.04		0.22
Poland	0.66	0.30	0.79	0.29	0.44	0.16	0.25
Turkey	0.27	0.27	0.22	0.21	0.12	0.12	0.20
Viet Nam	0.11	0.13	0.09	0.09	0.50	0.60	0.27
Romania	0.22	0.24	0.24	0.20	0.03	0.03	0.16
Venezuela	0.04	0.17	0.02	0.04	0.03	0.11	0.10
Mozambique	0.18	0.18	0.02	0.03	0.18	0.17	0.13
Nigeria	0.00	0.00	0.25	0.43	0.00	0.00	0.14
Germany	0.01	0.01	0.10	0.14	0.14	0.18	0.11
Pakistan	0.04	0.06	0.06	0.09	0.04	0.05	0.07

Supplementary Table 7. Oil-gas and Coal emissions in China in different studies.

	Oil and Gas		Coal	
	Prior ($Tg\ a^{-1}$)	Posterior ($Tg\ a^{-1}$)	Prior ($Tg\ a^{-1}$)	Posterior ($Tg\ a^{-1}$)
This study	1.2	2.2	21.1	20.2
	1.1	2.1	18.5	18.2
	3.2	3.8	19.9	18.2
Liang et a.[38]	1.2	1.8	16.6	18.0
	1.2	1.4	16.6	16.2
Chen et al.[39]	1.1	2.7	19.5	16.6

Supplementary Table 8. Posterior estimates of all sectors and comparison with another global TROPOIN inversion work.

	**This study**			Qu et al. [34]	
	Prior estimate	Percentage of prior emission covered by our 15 inversion domains	Posterior estimates	TROPOMI inversion	TROPOMI-GOSAT joint inversion
**Total sources**	516	71%	556	556	570
**Anthropogenic**	325	82%	363	336	363
Oil and Gas	48	96%	61	53	56
Coal	33	96%	33	NA	NA
**Livestock**	115	71%	127	126	139
Rice	37	86%	42	NA	NA
**Wastewater**	38	80%	42	44	44
Landfill	29	87%	31	27	31
Other anthropogenic sources	25	83%	27	23	26
**Natural**	191	51%	193	220	207
Wetlands	162[#]	50%	165	195	183
Termites	12	56%	13	12	12
Open fires	15	61%	13	11	10
Seeps	2	74%	2	2	2

Supplementary References

1. Shen, L. *et al.* Unravelling a large methane emission discrepancy in Mexico using satellite observations. *Remote Sensing of Environment* **260**, 112461 (2021).
2. Maasakkers, J. D. *et al.* Gridded National Inventory of U.S. Methane Emissions. *Environ. Sci. Technol.* **50**, 13123–13133 (2016).
3. Bloom, A. A. *et al.* A global wetland methane emissions and uncertainty dataset for atmospheric chemical transport models (WetCHARTs version 1.0). *Geosci. Model Dev.* **10**, 2141–2156 (2017).
4. Schuldt, K. N. & et al. Multi-laboratory compilation of atmospheric methane data for the period 1983-2021; obspack_ch4_1_GLOBALVIEWplus_v5.1_2023-03-08. *NOAA Earth System Research Laboratory, Global Monitoring Laboratory* (2023) doi:http://doi.org/10.25925/20230308.
5. Ma, S. *et al.* Satellite Constraints on the Latitudinal Distribution and Temperature Sensitivity of Wetland Methane Emissions. *AGU Advances* **2**, (2021).
6. Neininger, B. G., Kelly, B. F. J., Hacker, J. M., Lu, X. & Schwietzke, S. Coal seam gas industry methane emissions in the Surat Basin, Australia: comparing airborne measurements with inventories. *Phil. Trans. R. Soc. A.* **379**, 20200458 (2021).
7. Krings, T. *et al.* Quantification of methane emission rates from coal mine ventilation shafts using airborne remote sensing data. *Atmos. Meas. Tech.* **6**, 151–166 (2013).
8. Yacovitch, T. I. *et al.* Methane emissions in the Netherlands: The Groningen field. *Elementa: Science of the Anthropocene* **6**, 57 (2018).
9. Fiehn, A. *et al.* Estimating CH_4, CO_2 and CO emissions from coal mining and industrial activities in the Upper Silesian Coal Basin using an aircraft-based mass balance approach. *Atmos. Chem. Phys.* **20**, 12675–12695 (2020).
10. Zavala-Araiza, D. *et al.* Methane emissions from oil and gas production sites in Alberta, Canada. *Elementa: Science of the Anthropocene* **6**, 27 (2018).
11. Johnson, M. R., Tyner, D. R., Conley, S., Schwietzke, S. & Zavala-Araiza, D. Comparisons of Airborne Measurements and Inventory Estimates of Methane Emissions in the Alberta Upstream Oil and Gas Sector. *Environ. Sci. Technol.* **51**, 13008–13017 (2017).
12. Peischl, J. *et al.* Quantifying atmospheric methane emissions from oil and natural gas production in the Bakken shale region of North Dakota. *J. Geophys. Res. Atmos.* **121**, 6101–6111 (2016).
13. Peischl, J. *et al.* Quantifying Methane and Ethane Emissions to the Atmosphere From Central and Western U.S. Oil and Natural Gas Production Regions. *J. Geophys. Res. Atmos.* (2018) doi:10.1029/2018JD028622.
14. Karion, A. *et al.* Aircraft-Based Estimate of Total Methane Emissions from the Barnett Shale Region. *Environ. Sci. Technol.* **49**, 8124–8131 (2015).
15. Lyon, D. R. *et al.* Concurrent variation in oil and gas methane emissions and oil price during the COVID-19 pandemic. *Atmos. Chem. Phys.* **21**, 6605–6626 (2021).
16. Pétron, G. *et al.* A new look at methane and nonmethane hydrocarbon emissions from oil and natural gas operations in the Colorado Denver-Julesburg Basin. *J. Geophys. Res. Atmos.* **119**, 6836–6852 (2014).
17. Schwietzke, S. *et al.* Improved Mechanistic Understanding of Natural Gas Methane Emissions from Spatially Resolved Aircraft Measurements. *Environ. Sci. Technol.* **51**, 7286–7294 (2017).
18. Cui, Y. Y. *et al.* Inversion Estimates of Lognormally Distributed Methane Emission Rates From the Haynesville-Bossier Oil and Gas Production Region Using Airborne Measurements. *J. Geophys. Res. Atmos.* **124**, 3520–3531 (2019).

19. Barkley, Z. R. *et al.* Quantifying methane emissions from natural gas production in north-eastern Pennsylvania. *Atmos. Chem. Phys.* **17**, 13941–13966 (2017).
20. EDF. New Mexico oil & gas data. (2019), https://www.edf.org/nm-oil-gas/ (accessed August 2021).
21. Pétron, G. *et al.* Investigating large methane enhancements in the U.S. San Juan Basin. *Elementa: Science of the Anthropocene* **8**, 038 (2020).
22. Ren, X. *et al.* Methane Emissions from the Marcellus Shale in Southwestern Pennsylvania and Northern West Virginia Based on Airborne Measurements. *J. Geophys. Res. Atmos.* **124**, 1862–1878 (2019).
23. Foster, C. S. *et al.* Quantifying methane emissions in the Uintah Basin during wintertime stagnation episodes. *Elementa: Science of the Anthropocene* **7**, 24 (2019).
24. Peischl, J. *et al.* Quantifying atmospheric methane emissions from the Haynesville, Fayetteville, and northeastern Marcellus shale gas production regions. *J. Geophys. Res. Atmos.* **120**, 2119–2139 (2015).
25. Lu, X. *et al.* Observation-derived 2010-2019 trends in methane emissions and intensities from US oil and gas fields tied to activity metrics. *Proc. Natl. Acad. Sci. U.S.A* **120**, e2217900120 (2023).
26. Lu, X. *et al.* Methane emissions in the United States, Canada, and Mexico: evaluation of national methane emission inventories and 2010–2017 sectoral trends by inverse analysis of in situ (GLOBALVIEWplus CH_4; ObsPack) and satellite (GOSAT) atmospheric observations. *Atmos. Chem. Phys.* **22**, 395–418 (2022).
27. Zhang, Y. *et al.* Quantifying methane emissions from the largest oil-producing basin in the United States from space. *Sci. Adv.* **6**, eaaz5120 (2020).
28. Saunois, M. *et al.* The global methane budget 2000–2012. *Earth Syst. Sci. Data* **8**, 697–751 (2016).
29. Saunois, M. *et al.* The Global Methane Budget 2000–2017. *Earth Syst. Sci. Data* **12**, 1561–1623 (2020).
30. Turner, A. J. *et al.* Estimating global and North American methane emissions with high spatial resolution using GOSAT satellite data. *Atmos. Chem. Phys.* **15**, 7049–7069 (2015).
31. Zhang, Y. *et al.* Attribution of the accelerating increase in atmospheric methane during 2010–2018 by inverse analysis of GOSAT observations. *Atmos. Chem. Phys.* **21**, 3643–3666 (2021).
32. Lu, X. *et al.* Global methane budget and trend, 2010–2017: complementarity of inverse analyses using in situ (GLOBALVIEWplus CH_4 ObsPack) and satellite (GOSAT) observations. *Atmos. Chem. Phys.* **21**, 4637–4657 (2021).
33. Maasakkers, J. D. *et al.* Global distribution of methane emissions, emission trends, and OH concentrations and trends inferred from an inversion of GOSAT satellite data for 2010–2015. *Atmos. Chem. Phys.* **19**, 7859–7881 (2019).
34. Qu, Z. *et al.* Global distribution of methane emissions: a comparative inverse analysis of observations from the TROPOMI and GOSAT satellite instruments. *Atmos. Chem. Phys.* **21**, 14159–14175 (2021).
35. Fraser, A. *et al.* Estimating regional methane surface fluxes: the relative importance of surface and GOSAT mole fraction measurements. *Atmos. Chem. Phys.* **13**, 5697–5713 (2013).
36. Shen, L. *et al.* Satellite quantification of oil and natural gas methane emissions in the US and Canada including contributions from individual basins, *Atmos. Chem. Phys.*, **22**, 11203–11215, https://doi.org/10.5194/acp-22-11203-2022 (2022).
37. Zavala-Araiza, D. *et al.* A tale of two regions: methane emissions from oil and gas production in offshore/onshore Mexico. *Environ. Res. Lett.* **16**, 024019 (2021).

38. Liang, R. *et al.* East Asian methane emissions inferred from high-resolution inversions of GOSAT and TROPOMI observations: a comparative and evaluative analysis, *Atmos. Chem. Phys. Discuss.*, https://doi.org/10.5194/acp-2022-508 (2022).
39. Chen, Z. *et al.* Methane emissions from China: a high-resolution inversion of TROPOMI satellite observations, *Atmos. Chem. Phys.*, **22**, 10809–10826, https://doi.org/10.5194/acp-22-10809-2022 (2022).
